# High-throughput 16S rRNA gene sequencing of the microbial community associated with palm oil mill effluents of two oil processing systems

**DOI:** 10.1038/s41598-021-92513-4

**Published:** 2021-06-24

**Authors:** Benedicte Ella Zranseu Aka, Theodore N’dede Djeni, Simon Laurent Tiemele Amoikon, Jan Kannengiesser, Naaila Ouazzani, Marcellin Koffi Dje

**Affiliations:** 1 Laboratoire de Biotechnologie et Microbiologie des Aliments, Université Nangui Abrogoua, 02 BP 801 Abidjan, Côte d’Ivoire; 2 Institute IWAR, Faculty of Civil and Environmental Engineering, Technische Universität Darmstadt, Darmstadt, Germany; 3 Laboratory of Hydrobiology, Ecotoxicology and Sanitation (LHEA, URAC 33), Faculty of Sciences Semlalia, Cadi Ayyad University, Marrakech, Morocco

**Keywords:** Biotechnology, Ecology, Microbiology

## Abstract

Palm Oil Mill Effluents (POME) are complex fermentative substrates which habour diverse native microbial contaminants. However, knowledge on the microbiota community shift caused by the anthropogenic effects of POME in the environment is up to date still to be extensively documented. In this study, the bacterial and archaeal communities of POME from two palm oil processing systems (artisanal and industrial) were investigated by Illumina MiSeq Platform. Despite the common characteristics of these wastewaters, we found that their microbial communities were significantly different with regard to their diversity and relative abundance of their different Amplicon Sequence Variants (ASV). Indeed, POME from industrial plants harboured as dominant phyla *Firmicutes* (46.24%), *Bacteroidetes* (34.19%), *Proteobacteria* (15.11%), with the particular presence of *Spirochaetes*, *verrucomicrobia* and *Synergistetes*, while those from artisanal production were colonized by *Firmicutes* (92.06%), *Proteobacteria* (4.21%) and *Actinobacteria* (2.09%). Furthermore, 43 AVSs of archaea were detected only in POME from industrial plants and assigned to *Crenarchaeota*, *Diapherotrites*, *Euryarchaeota* and *Nanoarchaeaeota* phyla, populated mainly by many methane-forming archaea. Definitively, the microbial community composition of POME from both type of processing was markedly different, showing that the history of these ecosystems and various processing conditions have a great impact on each microbial community structure and diversity. By improving knowledge about this microbiome, the results also provide insight into the potential microbial contaminants of soils and rivers receiving these wastewaters.

## Introduction

Palm oil, squeezed from the fruits of the oil palm tree (*Elaeis Guineensis*) is the most widely used vegetable oil in the world. Grown only in the tropics, the plant is very productive and offers a far greater yield at a lower cost of production than other vegetable oils, thus standing up from them^1^. The oil from the palm tree is of high-quality and primarily used in developing countries for cooking, but also in food products, detergents, cosmetics and, to a small extent biofuel^2^. Plantations are spreading across Asia, Latin America and Africa; with Côte d'Ivoire, ranking second producers of palm oil in Africa, with an annual production of about 400,000 tons^3^ (CNUCED 2016). According to previous reports^4^, unlike in South-East Asia, where the processing of crude palm oil is entirely undertaken by agro-industries in high-technology well equipped mills, palm oil processing in most of African countries is undertaken by three distinct groups of actors: (i) traditional producers using basically manual methods with the use of rudimentary tools, (ii) small-scale (or non-industrial) producers using various low-efficiency machineries; and (iii) finally the industrial mills with technologically up-to-date machinery, established by agro-industrial complexes. It is also a recurring situation in Cote d’Ivoire, where the number of traditional producers is increasing, due to the strong demand for oilseed products and recent technological and scientific advances in this sector.

Palm oil worldwide production and demand is rapidly and intensively increasing; in consequence, its production results in the generation of large quantities of polluted wastewater commonly referred to as palm oil mill effluents (POME)^5^. Some studies estimated that a ton of crude palm oil production required 5 to 7.5 tons of water and at least 50% of this water (about 3.5 m^3^) end up as POME^6^. Wastewaters from such intensive agricultural activity typically have high concentrations of organic matters and nutrients. POMEs are viscous, brownish liquid containing about 95–96% water, 0.6–0.7% oil and 4–5% total solids, acidic (pH 4–5) with high organic content [Chemical oxygen demand (COD) 50,000 mg/L, biological oxygen demand (BOD) 25,000 mg/L]^7^; resulting in high pollutant loads. According to the standards on industrial effluents discharge in Côte d’Ivoire, the limit values are 300 mg/L for COD, 100 mg/L for BOD_5_ and the pH should be in the range of 6.5–8.5. Therefore, these wastewaters generate particular problems and challenges to producers, since high organic matter contents can lead to effluent management issues if they are allowed to discharge directly into receiving environments. For this reason, POME must be treated prior to disposal. However, when it comes to liquid waste management, most traditional and small-scale palm oil processors do not adhere to any environmental protection practices, while the environmental awareness level of the operators in this industrial area is low as reported in previous studies^8^. In addition, traditional processors operate so close to nature that they simply return liquids to the surrounding bushes. The discharged quantities are so small that the ground easily absorbs the waste matter and the operators have not yet seen their activities as injurious to their surroundings^8^, whereas in short and medium terms, the reverse effects on environment, in particular, on soils and waterways in which these effluents are discharged become visible.

Moreover, environmental and social concerns are amplified by the escalating expansion of oil palm plantations and uncontrolled processing in tropical areas. Undoubtedly, environmental contamination is a relevant problem which has a negative impact on human health until today. Many approaches have been used to monitor and reduce this problem but, it still remains a difficult issue. To ensure both humans and environment security against adverse effects of this pollution, a deep insight into the pollutant load, particularly the microbial communities of these effluents must be designed. Indeed, it has long been recognized that certain microbial groups in waste are responsible for breaking down various organic compounds and for the suppression of pathogens in waste^9^, while some could appear as potential bioindicators in the receiving waterways after final discharging^10^. In addition, if the physicochemical properties of POME are well documented, this is not the case for its microbiological aspects, which until then seem to have been little explored. This reveals a dearth of information on this microbiome and justifies the need for a thorough understanding of it. Consequently, the mastery of this microbiome lays a basis to promote a better understanding of the nature and types of microorganisms populating POME. Therefore, to ensure a sustainable practice of palm oil processing, it is essential to have more knowledge on the bacterial population, diversity and how they are related to the biodegradation process and/or severity of pollution of discharging water or soil. In this study, Illumina MiSeq sequencing was used to investigate the bacterial community composition of POME from two types of oil production (artisanal and industrial) in four geographical localities of Côte d’Ivoire. We compared the microbial community structures of POME samples with the aim to determine the abundance and diversity of bacterial communities and provide insight of the potential functional microorganisms able to achieve higher reduction of organic load present in POME and their potential level of discharging environment microbial pollution.

## Results

### Physicochemical characteristics of POME samples

The physicochemical characteristics of POME samples collected in four locations of palm oil production in Côte d’Ivoire are presented in Table 1. All the samples analysed were acidic with pH comprised between 4.11 and 4.74. However, the other parameters differed in the samples of both production types. Indeed, these samples were enriched in total solids (134,880 ± 42.61 and 144,180 ± 49.72 mg/L), in phenolics (441.7 ± 7 and 458.5 ± 14 mg/L) and in fatty matters (41,330 ± 52 and 42,750 ± 61.2 mg/L) respectively for Divo and Zouan Hounien, localities where traditional oil production is practiced.

**Table 1:** Characteristics of palm oil mill effluents (POME) from four localities in Côte d’Ivoire.

Parameters	Localities of palm oil production				Discharge effluent standards
	Aboisso^A^	Sikensi^A^	Divo^B^	Zouan houien^B^	
pH	4.16 ± 0.47^a^	4.11 ± 0.4^a^	4.25 ± 0.13^a^	4.74 ± 0.5^b^	6.5–8.5
Total solids	62,460 ± 59^a^	43,760 ± 108^a^	134,880 ± 42.6^b^	144,180 ± 49.7^b^	–
Phenolics	264.1 ± 8.3^a^	265.3 ± 5.3^a^	441.7 ± 7^b^	458.5 ± 14^b^	0.3
Oil and grease	8,500 ± 33^a^	28,950 ± 47.1^b^	41,330 ± 52^c^	42,750 ± 61.2^c^	30
COD	ND	ND	ND	ND	300
BOD_5_	ND	ND	ND	ND	100

All parameters are in mg/L except pH. Averages with the same letter on same line only for chemical characteristics are not significantly different by ANOVA (α = 0.05).

*ND* not determined.

^A^Industrial POME.

^B^Traditional POME. Each value is expressed as mean ± SEM (n = 3).

### Bacterial community structure and diversity

**Figure 1:**
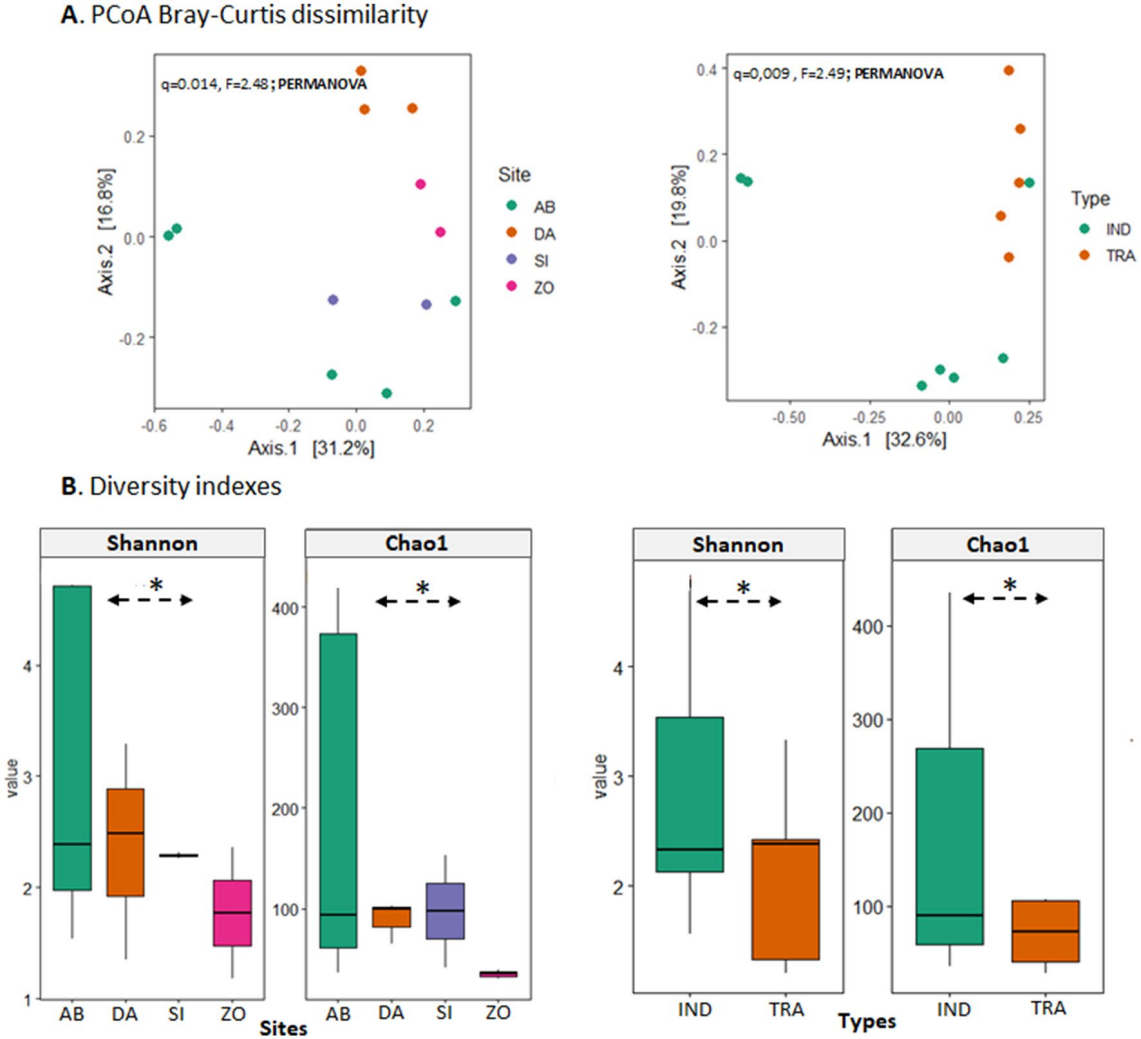
The overall bacterial community structure and diversity of palm oil mill effluents (POME) from Côte d’Ivoire. (**A**) PCoA using Bray–Curtis dissimilarity based on the genus-level ASV shows significant differences between samples based on palm oil processing areas (q = 0.014, F = 2.48, PERMANOVA) and the type of processing (q = 0.009, F = 2.49, PERMANOVA). (**B**) The boxplot shows the existence of similar microbial diversity (Chao richness and Shannon diversity) in the samples according to the area and the type of palm oil processing (**B**). The significance in the difference was calculated by Kruskal–Wallis test. Samples were collected in four distinct localities namely Aboisso (AB), Dabore (DA), Sikensi (SI) and Zouan Houien (ZO) by considering the two type of processing notably industrial (IND) and artisanal (TRA).

A total of 661,585 raw 16S V4–V5 sequences were obtained from twelve (12) out of 15 POME samples collected from four (4) localities of Côte d’Ivoire. The numbers of 16S V4–V5 sequences ranged from 32,448 to 74,934 among the samples, with samples from industrial oil production (e.g. AB3) having the highest number of bacteria ASV (439) and those of artisanal production (e.g. ZO3) the smallest number of ASV (30) (Table 2). The analysis of the overall bacterial community structure present in POME samples from the different production areas and with regard to both types of oil production (artisanal and industrial) was performed by PCoA using Bray–Curtis dissimilarity based on the species-level ASV relative abundance profiles, for visualizing the dissimilarities in community membership (Fig. 1A). The bacterial community structure in these POME samples, regarding the production areas significantly differed from one site to another (q = 0.01, F = 2.48, PERMANOVA). In the same way, the results also showed a distinct separation in the bacterial community structure of industrial oil (AB and SI) productions and artisanal ones (DA and ZO) (q = 0.01, F = 2.49, PERMANOVA).

**Table 2:** Alpha diversity analyses of the bacterial and archaeal communities in palm oil mill effluents (POME) samples.

Samples	Number of sequence	ASV	Pielou	Chao 1	Shannon	Simpson
AB1	58,756	66	0.48	69.17	1.99	3.52
AB2	45,984	93	0.54	90.4	2.39	4.28
AB3-2	48,842	375	0.79	379.02	4.69	39.41
AB3	60,492	439	0.79	448.72	4.79	44.13
AB4	74,850	38	0.44	35.25	1.56	2.42
SI1	74,186	42	0.61	49	2.27	6.38
SI2	74,934	68	0.47	158.46	2.34	3.32
DA1	68,531	110	0.71	107.5	3.33	15.05
DA2	49,329	68	0.31	73.16	1.32	1.82
DA3	36,172	101	0.53	106.16	2.42	4.89
ZO2	32,448	40	0.70	29	2.38	7.03
ZO3	37,061	30	0.32	41.13	1.21	1.97

Chao1 richness, Pielou evenness, Simpson’s and Shannon’s diversity indices were used to measure the species richness and evenness of ASV distribution within the bacterial community, with higher values of indices being regarded as higher genetic diversity in a production area or type. The observed community richness and diversity indices of the bacterial community are shown in Table 2. Based on these indices, the bacterial community of POME samples presented high richness and diversity. The range of Pielou indices was 0.31–0.79 while Chao1 richness ranged between 29 and 448.72. Shannon and Simpson indexes varied respectively between 1.21 and 4.79 and 1.97 and 44.13. For all indices, samples from industrial production in the South-East, particularly AB3 exhibited highest values while compared with the others. Indeed, the alpha diversity analysis (Chao species richness and Shannon diversity index) showed a high microbial diversity with regard to the sample origins and the type of palm oil production (Kruskal–Wallis test, P < 0.05) (Fig. 1B).

### Bacterial community taxonomic composition

**Figure 2:**
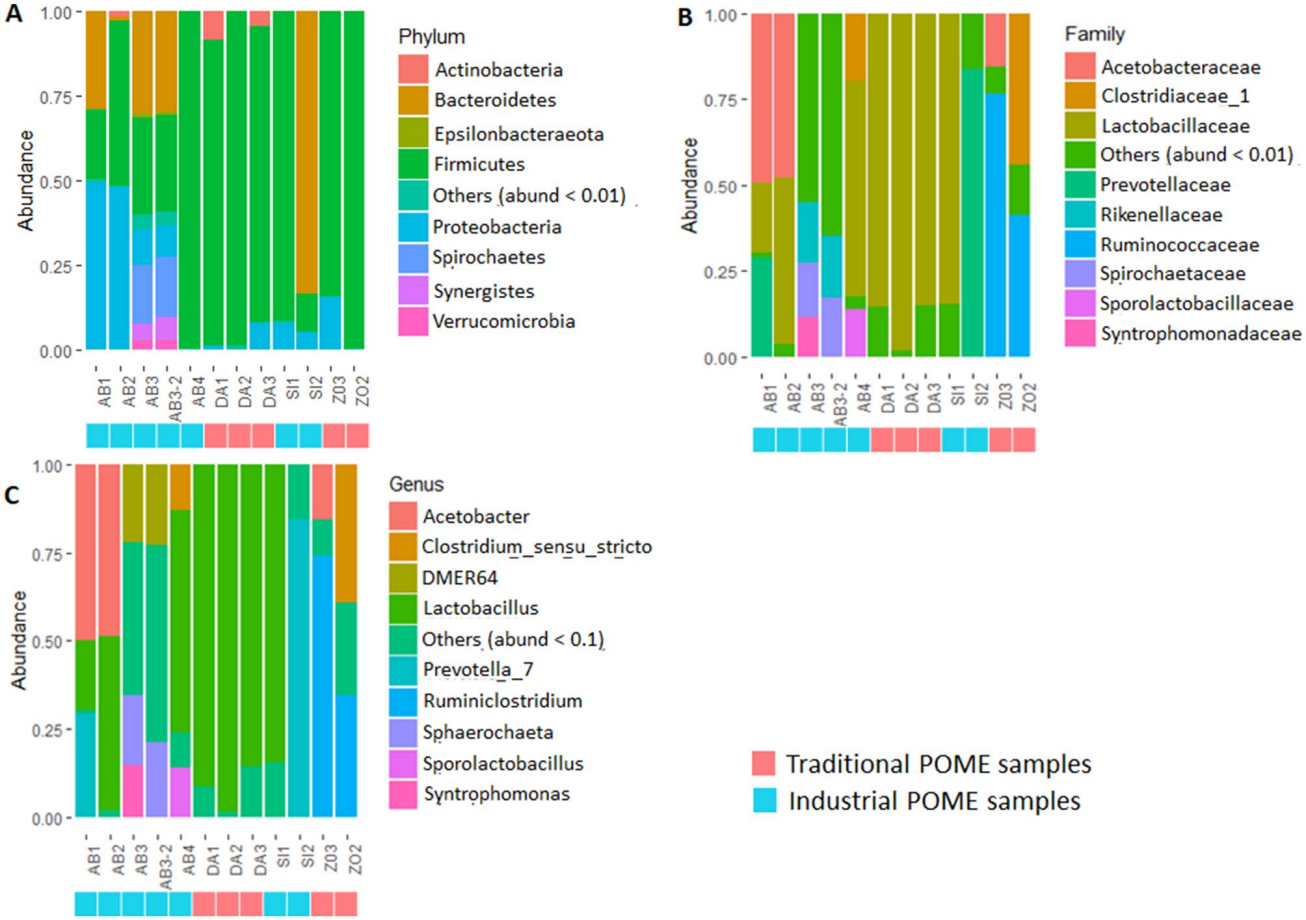
The taxon bar chart shows the relative abundance (%) of predominant bacteria at phylum level (**A**) family-level (**B**) and genus-level (**C**). The taxa with a mean relative abundance of less than 1% across the samples are combined and shown as others. The unclassified ASV were shown as NA.

The relative abundance at different taxonomic levels of the bacterial community composition for the twelve POME samples, notably phylum, family and genus is presented in Fig. 2. Globally, sequences were classified and assigned to 28 bacterial phyla and 103 families. “Rare Phyla” called others (phyla with a relative abundance ˂ 0.1%) account for 0.07%. ASV that could not be assigned were defined as unclassified microorganisms and accounted for 0.02%. Most of the traditional production samples and to a lesser extent some samples from industrial productions, especially those collected just at the factory output are dominated by *Firmicutes* with relative abundance percentages of at least 70%. The other samples of industrial production are either dominated by *Proteobacteria* with relative abundances of nearly 50% (AB1, AB2) or *Bacteroidetes* at more than 80% (SI1) (Fig. 2A). At the family level, *Lactobacillaceae*, belonging to *Firmicutes* phylum showed the highest relative abundance in most of POME samples, particularly in those from Daboré- Divo (DA), with an average relative abundance of 90.28%; except in ZO samples where dominance is ensured by *Ruminococaceae* (62.01%) and *Clostridiaceae* 1 (22.08%). This family was followed by other families from various phyla notably *Acetobacteraceae* (21.73%) and *Prevotellaceae* (8.10%) in AB samples, *Bifidobacteriaceae* (4.08%) in DA samples and *Prevotellaceae* (38.88%) in SI samples. Families classified as “others” with relative abundance ˂ 0.1% and the non-attributed families represented 1.45% and 0.63% respectively (Fig. 2B).

**Figure 3:**
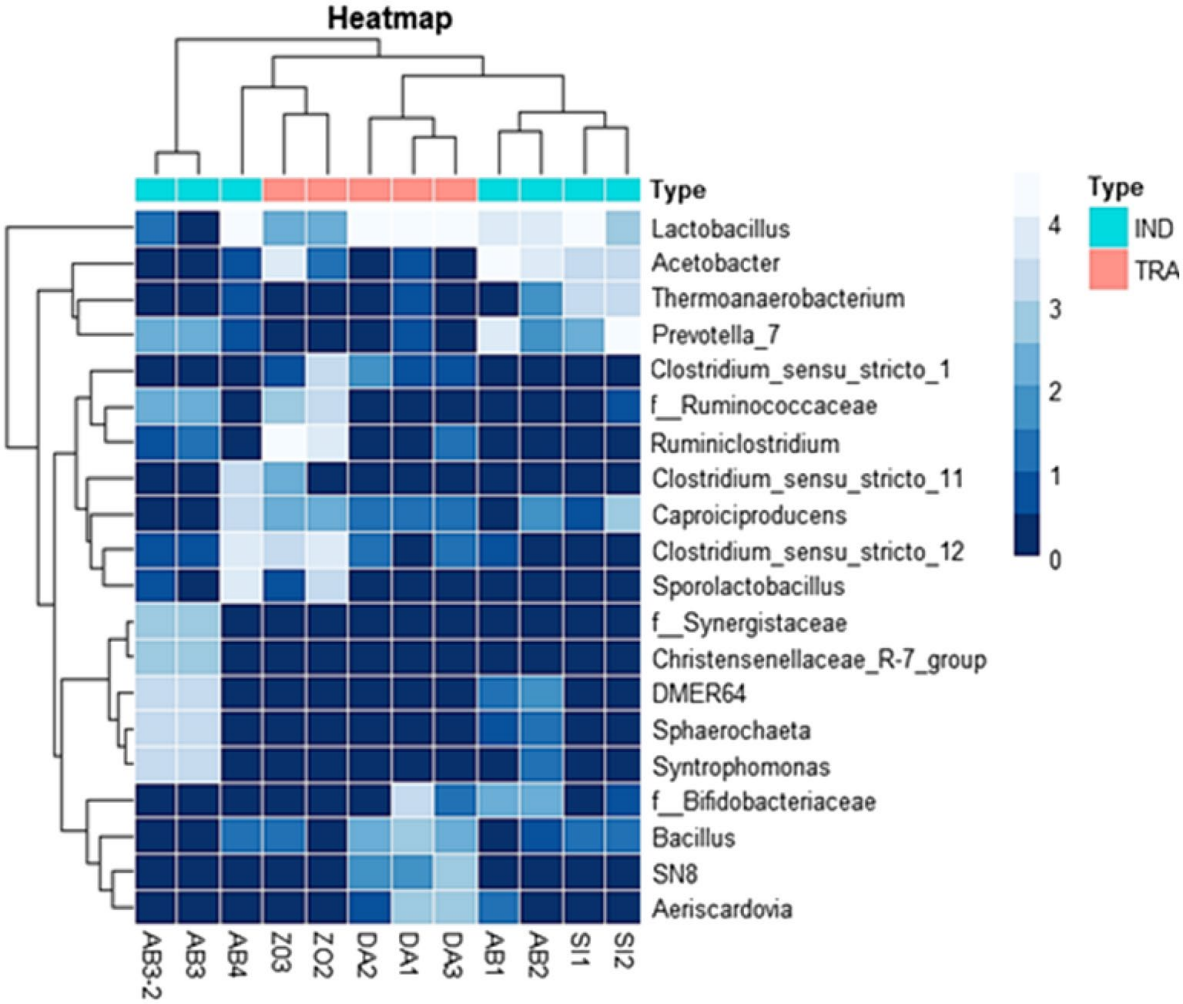
A hierarchically clustered heat map shows the bacterial genus-level differential abundance in the two types (industrial and artisanal) of palm oil processing in Côte d’Ivoire. The significantly top 20 bacterial genera between the processing types clustered here. The ASV relative abundance data were normalized [log10 (xi + 1)-transformed] before generating the heat map using R version 3.6.3 software (https://cran.r-project.org/bin/windows/base/old/3.6.3/). The abundance difference is shown as a color key with a blue colour gradient. The sample distribution of the different palm oil processing types over the clusters is shown above the heat map.

A total of 161 genera were detected among which 4.5% were unclassified. *Lactobacillus* (44.14%), *Prevotella* (15.01%), *Acetobacter* (10.39%), *Ruminiclostridium* (7.45%), *Clostridium sensus stricto* (5.07%) and *Sporolactobacillus* (1.94%) were on average, the relatively abundant genera in POME samples. The others with a relative abundance ˂ 0.1% represented 13.12% of the total ASV (Fig. 2C). A clustered heat map based on the relative abundance of the twenty predominant bacterial genera between different POME samples is showed in Fig. 3. As shown in this figure, samples from the same type of oil production, particularly, those of artisanal oil productions are clustered together. These samples harboured *Lactobacillus*, *Clostridium *sensu stricto 12, *Bacillus*. Samples from industrial oil productions were divided into two clusters containing on one hand, most of AB samples characterized by the presence of *Synergistaceae*, *Christensenellaceae*, *DMER64*, *Sphaerochaeta* and *Syntrophomonas*, and on the other hand, samples from Sikensi (SI) and Aboisso (AB) which were populated by *Lactobacillus*, *Acetobacter*, *Thermoanaerobacterium* and *Prevotella* (Fig. 3).

### Archaea community taxonomic

**Figure 4:**
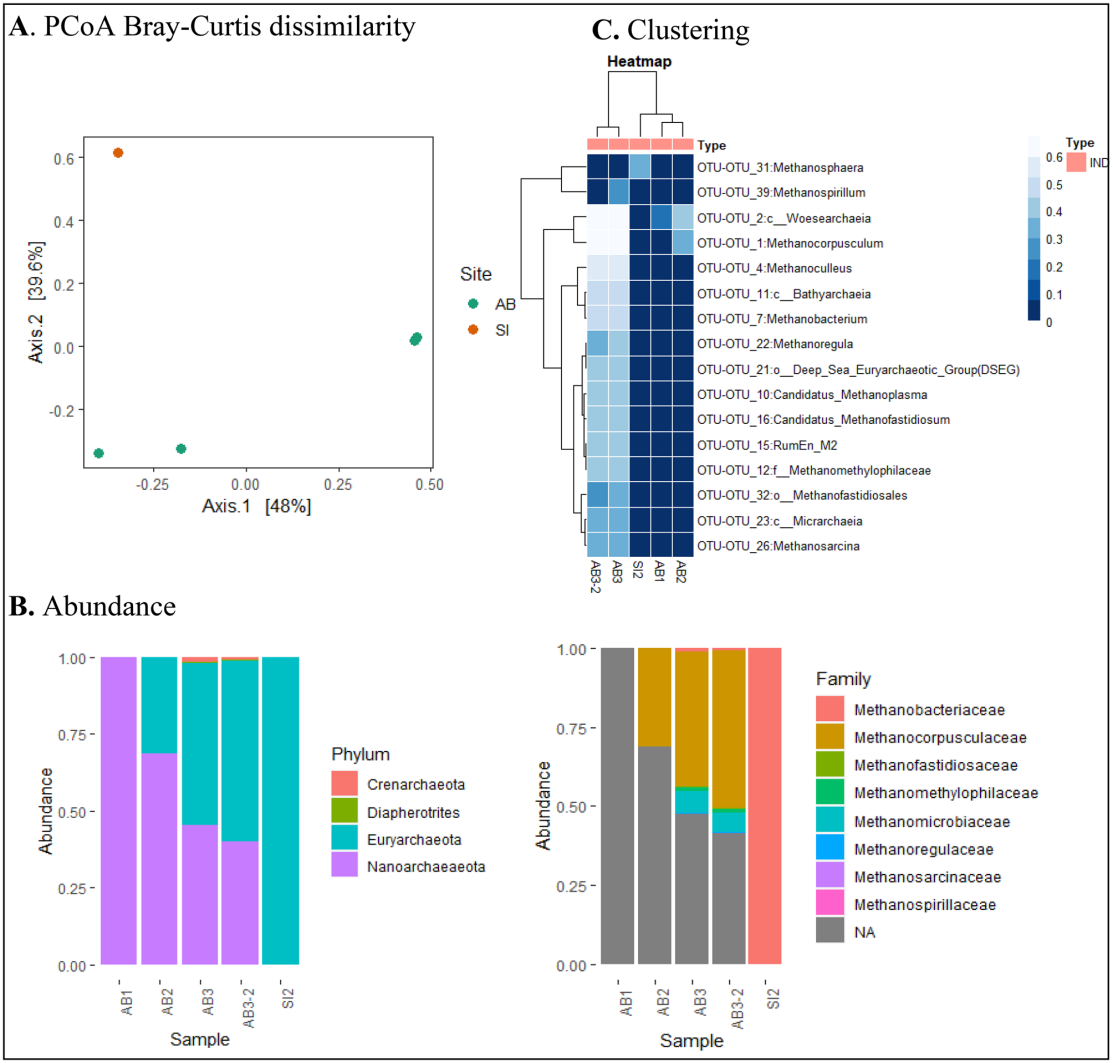
The overall Archaea community structure, relative abundance and clustering of palm oil mill effluents (POME) from Côte d’Ivoire. (**A**) PCoA using Bray–Curtis dissimilarity based on the genus-level ASV shows significant differences between samples (q = 0.014, F = 0.009, PERMANOVA). (**B**) Relative abundance (%) of predominant bacteria at phylum level. (**C**) The hierarchically heat map clustered with top 20 archaea genera between the positive samples. The abundance difference is shown as a color key with a blue colour gradient. The ASV relative abundance data were normalized [log10 (xi + 1)-transformed] before generating the heat map using R version 3.6.3 software (https://cran.r-project.org/bin/windows/base/old/3.6.3/).

The primers used for bacteria V4–V5 variable region sequencing have the ability to recover sequences from archaea. Thus, 43 ASV of archaea were recognized in 5 out of 12 samples provided by industrial processing type, namely AB1, AB2, AB3, AB3-2 and SI2. Table 3 and Fig. 4A summarize the number of taxa observed and the overall archaeal community structure in these samples. Sample AB3 had the highest number (38) of ASV among which 10 were distinct. Furthermore, the phyla *Crenarchaeota*, *Diapherotrites*, *Euryarchaeota*, *Nanoarchaeaeota* were identified (Fig. 4B). All of them were present in samples AB3 and AB3-2 while only *Nanoarchaeaeota* was identified in samples AB1 and *Euryarchaeota* in samples SI2. Two phyla, notably *Euryarchaeota* and *Nanoarchaeaeota* were observed in samples AB2. At the family level, *Methanocorpusculaceae* represented 45.55% of total ASV followed by *Methanomicrobiaceae* (6.45%), *Methanomethylophilaceae* (1.3%), *Methanobacteriaceae* (1.1%), *Methanofastidiosaceae* (0.35%), *Methanoregulaceae* (0.25%) and *Methanospirillaceae* (0.14%) on average (Fig. 4B). The non-attributed family ASV represented 44.86%. The heatmap constructed at the genus level indicates three clusters (Fig. 4C). The first one contains samples AB1 and AB2 that harboured *woesearchaeia* and *Methanocorpusculum*. Sample SI2, the single member of the second cluster, was populated by *Methanosphaera*. Samples AB3 and AB3-2 of the last cluster were characterized by the presence of all identified genera except *Methanosphaera*.

**Table 3:** Comparison of number of identified archaea taxa obtained from palm oil mill effluents (POME) samples.

Samples	AB1	AB2	AB3	AB3-2	SI2
Phylum	1	2	4	4	1
Order	0	1	6	6	1
Family	0	1	7	7	1
Genus	0	1	7	8	1
Numbers of ASV	1	3	30	38	1
Distinct ASV	0	0	3	10	1

## Discussion

POME samples of the present study were characterized by a low pH and high phenolics, total solids (TS) and fatty matters contents, which were higher than the discharge effluent standards in Cote d’Ivoire^11^. Similar acidic pHs were found in POME from Malaysia and Nigeria. However, for oil & grease, TS, and phenolics content, results obtained in these countries were lower^12,13^. Technical methods of oil extraction and oil palm trees varieties could be the reasons of these differences. POME contain significant amounts of carbohydrates, proteins, nitrogenous compounds, lipids, minerals, cellulose, hemicelluloses and lignin; so that they can be considered naturally as a fermentation medium for value-added products^14^. They are essential habitat for diverse microbes with a biotechnological importance like lipolytic and cellulolytic bacteria and wastewater treatment agent for example^5^. In this study, Next Generation of Sequencing was performed to improve the understanding of the bacterial community structure of POME from both artisanal and industrial oil productions in four different localities in Côte d’Ivoire. Recently, many studies have investigated the dynamics and diversity of microbial communities from different wastewater treatment systems by using high-throughput sequencing, which enables the detection of massive microbial populations^15–17^. Illumina MiSeq, in particular, has been successfully used to study various microbial communities from host-associated and free-living environments^18^. A thorough understanding on the microbial community structure of POME has also been carried out in various bioreactor configurations^19^. In the present study, various alpha diversity indexes (Shannon diversity and Chao-1 richness) were used to measure the species richness and diversity within the bacterial community of POME, where higher values of indices can be regarded as higher genetic diversity at a production area or type. In general, POME from industrial oil processing showed the highest number of ASV and alpha diversity indices, particularly some samples (AB3, AB3-2) from Aboisso in the South, which displays a higher bacterial diversity compared to the others. In this type of production, effluents mentioned above are all put to economically useful purpose through a biotreatment process. Thus, the intrinsic variation in microbial parameters within this production site would be based on nutrient availability and microbial enrichment over time, from the factory outlet, its stay through the lagoon station until its biodegradation in the digestion tank. This is totally different from the practices in the other types of production where effluents, once produced have just enough time to cool before being poured into nature, without allowing prior microbial enrichment. The observations made in this study were supported by other studies^20^ who similarly stated that the system performance and the microbial community of the activated sludge were different based on the operating conditions. In the same way, some authors suggested that bacteria playing important roles in the biodegradation at the early phases might become excessively dominant, hence modifying the diversity indices^10^. In addition, the high organic matter concentration, readily available to most bacteria could contribute to the increment of the bacterial population^21^, hence reducing the Shannon and Eveness indices in the early phases of POME treatment.

**Figure 5:**
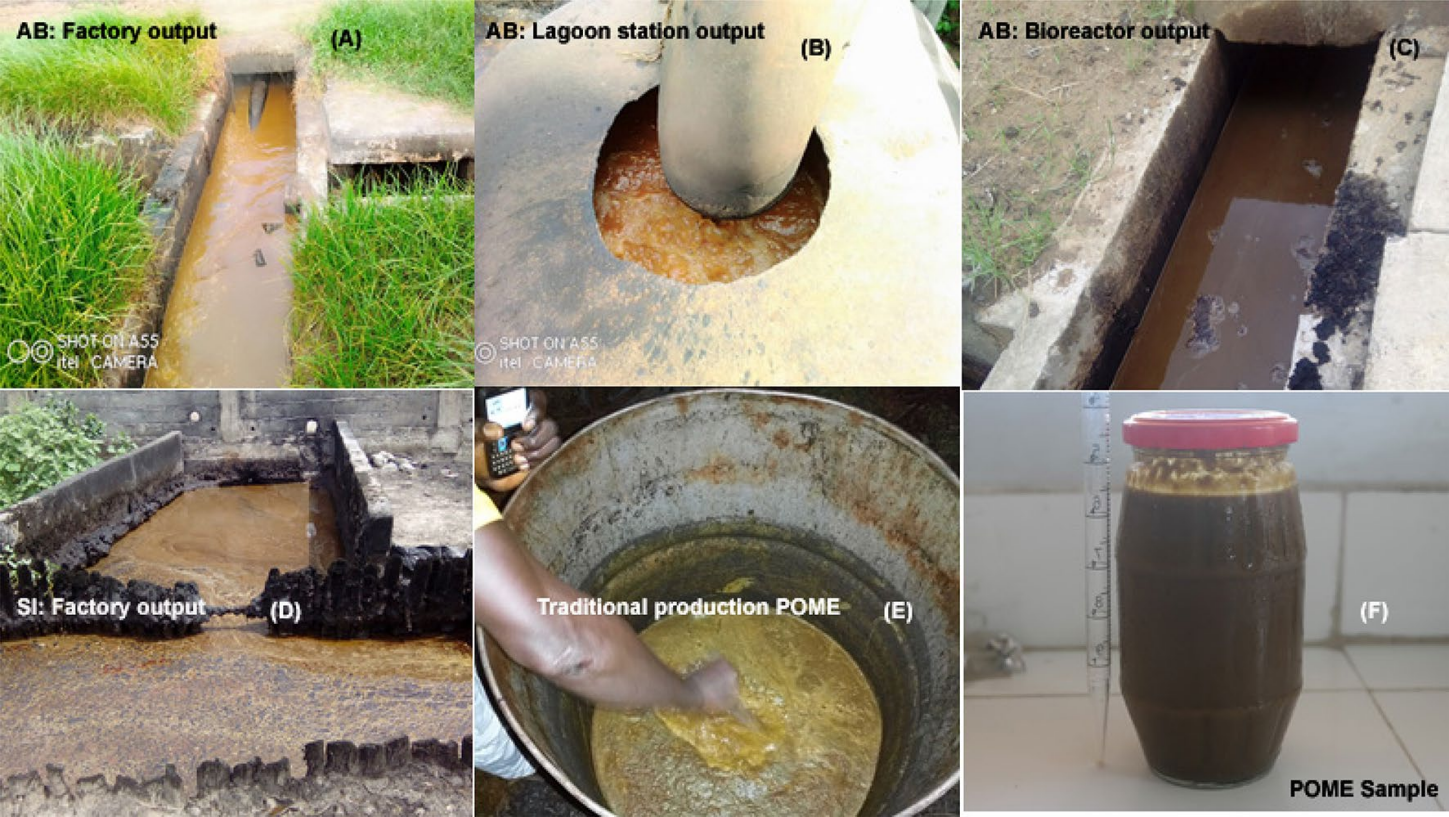
Palm oil mill effluents (POME) collection at various stages of processing in industrial plants and traditional production.

Statistical analysis of alpha diversity indices by Kruskal–Wallis test however revealed that significant differences in both richness and diversity indices were observed among communities from different localities and from both types of oil production; indicating that geography and oil production method would have impacted the microbial richness and evenness of POME. In addition, the richness and evenness indices obtained in this study were higher than those of previous studies from other oil production countries such as Malaysia^22^. This suggests that POME from Côte d’Ivoire would contain a high microbial diversity with a large reservoir of genetic variability. Besides, a significant difference of the bacterial community structure was observed according to the production areas and the type of palm oil production. Multivariate analyses through PCoA using Bray–Curtis dissimilarity and clustered heat map performed with the top 20 bacterial OTUs indicated that microbial community structures of samples from the same type of palm oil processing and the same locality were closer, except those from Aboisso located in the South-East of Côte d’Ivoire. Such discrepancy, particularly in samples from Aboisso seems obvious, due to the discrepancy in the sample collection points. Indeed, as previously reported^23^, the microbial structural differences between samples of various regions can be attributed to geographic location associated to other environmental factors such as elevation, climate, temperature, oxygen level, atmospheric pressure, sunlight, and length of sunlight radiation which may play roles in modulating the microbiome. However, this justification is not valid in the case of this study, at least for the first two factors mentioned. Indeed, statistical analyses by Mantel test showed that the species Bray–Curtis dissimilarity matrix did not have a significant relationship with environmental parameters of POME samples in one hand (Mantel statistic r: 0.429, p value = 0.333) and with the geographical separation of samples (Mantel statistic R: 0.6, p value = 0.25) in the other hand. Thus, POME from each production type or area might have been subjected to different kinds of external factors and environment stress along the final discharge exposure which potentially promoting microbial changes and affecting microbial community shift. In fact, in artisanal production areas, in particular ZO and DA, there are no post-production management methods for effluents before their final disposal. So once produced, these effluents just have a short time (maximum 48 h) to cool in tanks (Fig. 5E) before being eliminated. In this practice, effluents are not subjected to other sources of contamination or microbial multiplication other than the storage material and contact with ambient air; which therefore does not allow a consequent enrichment of these effluents in microorganisms. This is not the case for effluents from industrial productions, which during their long discharging circuit (circulation in open pipes over about 1 km), are subject to the effects of climate on the one hand and human and animal pressure on the other hand. The dispersion of samples from industrial productions, particularly those from Aboisso is also favoured by the presence of an anaerobic effluent treatment device which comprises a settling tank, a digester and a system for recovering the treated water. The supplementation of digestion step with inoculum provided by cow dung explains the particular grouping of samples collected at this level (AB3 and AB3-2).

Industrial production POMEs bacteria harboured *Firmicutes, Bacteroidetes*, *Proteobacteria*, *verrucomicrobia* and *Synergistetes* as major bacterial group in decreasing order, while POMEs from artisanal productions were colonized by *Firmicutes*, *Proteobacteria*, *Bacteroidetes* and *Actinobacteria*. POME microbiota is believed to play key roles in nutrient cycling, and these communities may adapt to local light conditions and nutrient availability^24,25^. Not all bacterial phyla varied in a similar fashion or magnitude across the sampled locations. The groups that varied stronger, notably *verrucomicrobia*, *Actinobacteria* and *Synergistetes,* might be more sensitive to local or regional micro environmental factors or growth media composition than other microbial groups, with a more uniform presence across the various areas or production types. The dominance of *Firmicutes*, *Bacteroidetes* and *Proteobacteria* in POME was consistent with previous reports on POME^22,26^ and other wastewaters or solid wastes^27,28^. In all POME samples dominated by the *Firmicutes* phylum, *Lactobacillaceae* family, with its main representative genus *Lactobacillus* was the major sub-taxa of this phylum (except samples from Zouan Hounien dominated by *Ruminococcaceae* with genus *Ruminoclostridium*), as their presence was associated to the acidic characteristic of POME the by production of lactic acid^22^. Some authors linked their origin to the remaining debris of palm fruit mesocarp and the oil palm empty fruit bunch generated during the crude palm oil extraction process^29,30^. In addition, these microorganisms have robust stabilities with respect to the oxygen and their capacity to generate a series of antimicrobial compounds that do compromise the viability of other bacteria, thus making them the dominant groups of these effluents. Moreover species of *Lactobacillus* genus are ubiquitous in the environment, and so have been found even in sewages, which implies an extraordinary adaptability with respect to negative physical–chemical factors, similar to those of POME and a response capacity that has not been assessed sufficiently^31^. In addition, *Lactobacillus* genus is a heterogeneous group of lactic acid bacteria (LAB) with important implications in wastewater treatment and in food and feed biotechnology. Moreover, the particular dominance of *Ruminiclostridium* genus in POME from the western Côte d’Ivoire (Zouan hounien) could be related to the specific richness of these effluents in total solids, as members belonging to *Ruminococcaceae* family includes species able to produce a wide variety of carbohydrate-active enzymes for enhancing cellulosic biomass degradation. And this is useful for the conversion of lignocellulosic biomass of POME to valuable products, such as transportation biofuels^32^.

The other dominant phyla *Bacteroidetes* and *Proteobacteria* were mainly represented in industrial production POME, respectively by *Prevotellaceae* and *Acetobacteraceae* families. *Prevotellaceae* family members were described as saccharolytic fermentative anaerobes involved in acidogenesis and promoted acetic acid production during anaerobic POME treatment^22,33^, explaining their massive presence in samples collected in the bioreactor tank in Aboisso (AB). Many of them were related to the organisms involved in the pollutant degradation, which suggest their importance for wastewater treatment^22^. *Proteobacteria* play a central role in the biological processes involved in the removal of organic matter and nitrogen in the ecoditch systems, but the distributions of subdivision may differ depending on the ambient environment, its salinity, and whether they are facing aerobic or anaerobic conditions^34^. Some members of *Prevotellaceae* family are lipase producer^35^, an important feature for oil and grease degradation in wastewaters and thus could fully contribute to reduce the fat which is abundant in POME from traditional oil production. Acetic acid bacteria (AAB) from *Proteobacteria* phylum are widely distributed in acidic environments^36^, thus justifying their presence in POME, an acidic microbiome, notably in AB samples which were more acidic. Members of *Acetobacteraceae* family are characterized by their potentiality to oxidize alcohols and sugars into acetic acid and strains of the genus *Acetobacter,* the most representative taxon of this phylum are referred to as decaying bacteria, responsible for the degradation of different substrates^37^.

The distributions of archaeal abundance in POME were poorly understood, which increased difficulty in the interpretation of this study. Indeed, they were exclusively detected in POME from industrial oil processing. Archaea could exist in extreme environments, such as the hot spring and acidic environment^38^, but also in the digestive system of humans and animals. They consist of halophiles, thermacidophiles and methanogens and play quite important role in converting or removing carbon, nitrogen, phosphorus, and other pollutants into environmentally friendly materials^39^. Four archaeal phyla, namely *Crenarchaeota*, *Diapherotrites*, *Euryarchaeota*, *Nanoarchaeaeota* were found in this study. *Nanoarchaeaeota* and *Euryarchaeota* were the only phyla detected at the factory outlet for both industrial production locations (AB and SI), highlighting a potential geographical influence instead of the similar chemical characteristics of POME. Previous studies reported the phylum *Euryarchaeota* as predominant archaea in municipal anaerobic sludge digesters^40^. Throughout the entire treatment process in AB, POME was enriched in supplementary phyla, notably in *Euryarchaeota* at the lagoon station output (AB2) and in *Euryarchaeota, Crenarchaeota* and *Diapherotrites* at the bioreactor step (AB3, AB3-2), probably due to animal farming surrounding the lagoon station and the use of cow dung as inoculum in the biorector. This was consistent with previous studies in which archaea, notably *Methanosarcina* species were reported to dominate in a process treating cattle manure^41^. Most of the family taxa from these phyla identified in POME appeared to be methanogen archaea. Indeed, it has been reported that, methanogens or methane-forming archaea are the most important in wastewater treatment plants^42^.

## Conclusion

The present study reported on the bacterial and archaeal communities of POME obtained from two types of palm oil processing in four geographical localities. The specific details of bacteria and archaea populating these wastewaters were depicted. We found that industrial production POMEs harboured as dominant bacteria phyla *Firmicutes*, *Bacteroidetes and Proteobacteria* and archaea communities dominated by methane-forming species; while POMEs from artisanal production were colonized by *Firmicutes* and *Proteobacteria*. Definitively, the microbial community composition of POME ecosystems in both industrial and artisanal plants was markedly different, showing that the history of these ecosystems and several potential factors including post production practices and micro environmental conditions have a great impact on their community structure and diversity. Thus, this work represents a step forward for providing valuable insight into the microbiology of these polluting wastewaters. Further research allowing to understand the enzymatic machinery and the bioremediation capacities of these microorganisms may offers a good opportunity to solve the environment problem linked to palm oil mill wastewater in Cote d’Ivoire.

## Materials and methods

### Sampling sites and sample collection

In Côte d’Ivoire, different areas, going from South-East to the West are famous for the cultivation and the processing of palm seeds into palm oil. Two types of processing (artisanal and industrial) are implemented either by companies or individuals, treating before discharging or directly discharging their effluents into receiving stream, ground water, and soil. Thus, samples were collected taking into account both types of processing in four localities notably Zouan Houien (6° 55′ 00′′ N, 8° 13′ 00′′ W) and Dabore (5° 49′ 59.999″ N 5° 22′ 0.001″ W) for artisanal processing in the western, and Sikensi (5° 40′ 34.021″ N 4° 34′ 32.902″ W) and Aboisso Ayenouan (5° 22′ 13″ N, 3° 19′ 47″ W) for industrial processing in the southern. Permission for POME sampling was obtained from the Centre Ivoirien Anti Pollution (CIAPOL), the relevant national institution dealing with pollution issues.

For the production areas with an effluent treatment station, in particular for Aboisso (AB), different collection points have been identified for samples collection (Fig. 5). These are the factory outlet (AB4, AB1), the lagoon station (AB2) and the bioreactor (AB3, AB3-2). For the second industrial producer who does not have any treatment station, three sample collection points, from the factory outlet to the final disposal were chosen. For artisanal productions, three producers were identified by localities for samples collection. Thus, a total of 15 samples collection points were defined, distributed as follows: 5 for Aboisso (AB), 3 for Sikensi (SI), 3 for Dabore (DA) and 3 for Zouan Hounien (ZO). Three POME samples of 500 ml each were collected at each selected point using a long vertical tube (150 cm) and kept in previously sterilised glass bottles that had been rinsed with effluents from the corresponding sample. Samples collected were then kept at 4 °C and directly transported at the laboratory where they were processed immediately.

### DNA extraction, PCR and illumina MiSeQ library preparation and sequencing

Three POME samples from each collection point were extracted and pooled to obtain a complete microbial community information. Then metagenomic DNAs were extracted from 2 ml of decanted POME sample mix using E.Z.N.A. soil DNA kit (OMEGA bio-tek, Georgia, USA) following the manufacturer’s instructions. DNA was stored at − 20 °C until further processing. For accessing POME microbiota, the V4–V5 hypervariable region of 16S rRNA gene was amplified using primers 515F (5′-GTGYCAGCMGCCGCGGTAA-3) and 926R (5-CCGYCAATTYMTTTRAGTTT-3)^43^. The complete reagent mixture (25 μl) contained 30 ng of sample DNA, 1X Phusion HF Buffer, 0.2 mM dNTPs mix, 0.2 μM each primer, 3% DMSO, 0.5 U Phusion Hot Start II DNA pol. and molecular Grade H_2_O. The PCR amplification conditions were as follows: an initial denaturation at 94 °C for 3 min followed by 35 cycles of 94 °C for 45 s, 50 °C for 60 s, and 72 °C for 90 s, and a final extension at 72 °C for 10 min. Resulting amplicons were cleaned, pooled, quantified and the sequencing was performed with Illumina MiSeq PE 300 following the procedures of genomics platform of CERMO FC (Montreal, Quebec, Canada).

### Data processing and analysis

Raw sequences from all samples analysed were demultiplexed and adapters and barcodes were removed. Then, the sequences quality was controlled using FastQC v0.11.5 according to a previously established procedure^44^. Data files obtained were imported and processed in the R-environment^45^ using various codes and inbuilt functions available in different R-packages. These files were filtered and trimmed using the filterAndTrim() function of the dada2 package^46^ and bases with low-quality scores were discarded. DADA 2 (Divisive Amplicon Denoising Algorithm) pipeline was used for processed the filtered data files. The steps of dereplication, core denoising algorithm and merging of the base pairs were carried out to obtain the full denoised sequences. Then, the removeBimeraDenovo() function of the dada2 package was used for removing chimeras. ASV sequences were assigned to a taxonomy using the Silva (Silva_nr_v132) database^47^ with assignTaxonomy() function of the same dada2 package and a phyloseq data object was created. A phylogenetic tree for the taxa was constructed using the R-package ape^48^ using the prune_taxa() functions of the phyloseq package in R^49^.

### Statistical analysis

The phyloseq object was used to calculate microbial abundances, α, β diversity analysis and to carried out statistical tests from various R packages^49,50^ after rarefication. The rarefaction depth was 12,321 reads per sample. Plot for relative taxa abundances was made using the microbiome package^51^ using the entire 16S dataset. Chao1, Pielou, Simpson and Shannon index estimates of alpha diversity were measured from each sample using the phyloseq package. Kruskal–Wallis test was performed among sample categories while measuring Shannon and Chao1 index. Principal Coordinate Analysis (PCoA) was performed using Bray–Curtis dissimilarity matrix^52^ between samples and visualized using the phyloseq package^49^. Stratified permutational multivariate analysis of variance (PERMANOVA) with 999 permutations was conducted on all PCoA with the vegan package to observe the statistical significance of clusters according to the sample categories. To assess the statistical significance (p < 0.05) p-values for multiple comparisons were adjusted according to the Benjamini and Hochberg method to control False Discovery Rate^53^ while performing multiple testing on taxa abundance according to sample categories. For heatmap analysis, data was log-transformed (log x_i_ + 1) and the plot was done from top 20 ASV using microbiome R package^51^. All data were analysed in R version 3.6.3. And finally, mantel test was performed to assess correlations between species abundance and environmental parameter on the one hand and between species abundance and geographic distance on the other hand, using vegan and geosphere packages in R.

## Data Availability

The sequence data generated are publicly available in the NCBI SRA database under the accession numbers PRJNA656100 (https://www.ncbi.nlm.nih.gov/sra/PRJNA656100).
